# Quality of Bovine Chilled or Frozen-Thawed Semen after Addition of Omega-3 Fatty Acids Supplementation to Extender

**Published:** 2013-09-18

**Authors:** Abbas Abavisani, Javad Arshami, Abbas Ali Naserian, Mohammad Ali Sheikholeslami Kandelousi, Mohammad Azizzadeh

**Affiliations:** 1 Division of Physiology, Department of Basic Sciences, Veterinary Faculty, Ferdowsi University of Mashhad, Mashhad, Iran; 2 Embryonic and Stem Cell Biology and Biotechnology Research Group, Institute of Biotechnology, Ferdowsi University of Mashhad, Mashhad, Iran; 3 Department of Animal Sciences, Faculty of Agriculture, Ferdowsi University of Mashhad, Mashhad, Iran; 4 Department of Clinical Sciences, Faculty of Veterinary Science, Ferdowsi University of Mashhad, Mashhad, Iran

**Keywords:** Bovine, Semen Analysis, Omega-3 Fatty Acids, Chilling, Freezing

## Abstract

**Background:**

This study was conducted to evaluate the potential protective effects of omega-3 poly unsaturated fatty acids (Ω-3 PUFAs) on bovine sperm quality in response to cooling and cryopreservation.

**Materials and Methods:**

In this experimental study included ejaculates from five proven fertile bulls, allocated to the control and the four experimental groups. For group 1, polyethylene glycol (PEG) as a solvent was added alone to the extender, while for groups 2, 3 and 4, different concentration of omega-3 PUFAs (1, 2.5 and 5%, respectively) in combination with PEG were added to the semen extender. Motility [using computer aided sperm analysis (CASA)], viability and morphology of bovine sperm were investigated after 24 and 48 hours in both cold liquid storage and frozen-thawed conditions.

**Results:**

Our findings showed that PEG has some detrimental effects on sperm quality. Cooling as well as cryopreservation decreased significantly most of measured variables of sperm as compared to fresh semen, whereas the treatments did not improve sperm quality. Furthermore, levels of some variables were decreased significantly during treatments (p<0.05).

**Conclusion:**

Addition of Ω-3 PUFAs to semen extenders cannot be effectively introduced to conservation media as well as sperm membrane in order to protect spermatozoa in response to cooling and freezing. It can be suggested if Ω-3 PUFAs is supplemented to the diet of bulls in order to modify the fatty acid compositions of sperm, they might perform their preventive properties.

## Introduction

The demand for semen from bulls of high genetic merit has improved developing and refining storage technologies for cattle semen. In previous decades, there have been many improvements in the methodological approach of cooling and freezing of spermatozoa. Due to the routine incorporation of egg yolk or milk with glycerol as agents to protect the spermatozoa during cooling and freezing procedures, some progress has been achieved regarding biochemical environment and physical conditions in order to improve sperm cryopreservation (11). The main storage technologies, including liquid stored and frozen-thawed semen, are considered to be applicable methods due to relative advantages and disadvantages in both procedures (2).

Cooling and freezing procedures result in a number of physical changes in the external environment of cell suspension such as water and solute movement (3). In frozen-thawed semen, there is a significant decline in motility, viability and forward progression in the female reproductive tract which causes a reduction in fertility (4). Despite many hypotheses, the exact molecular mechanisms responsible for decreased sperm fertility during *in vitro* storage remain unclear. However, evidence is accumulating that this reduced fertility is related to the disruption and damage of the sperm membrane (5). Functional characteristics of spermatozoa are extensively affected by the lipid composition of the sperm membrane (6-10).

The extent of membrane damage may include a variety of different conditions, like changes in the organization, fluidity, permeability, lipid composition of the membrane bilayer, and total membrane disruption (11). In addition, the cryopreservation process induces reactive oxygen species (ROS) (12). The lipid per-oxidation by ROS results in a loss of polyunsaturated fatty acids from the plasma membrane and a decrease in sperm survival and fertility (13). 

Phospholipid composition of sperm, in particular its high content in omega-3 polyunsaturated fatty acids (Ω-3 PUFAs), maintained plasma membrane fluidity and integrity, crucial factors for sperm fertility (14). The differences between species in the susceptibility of spermatozoa to cooling, freezing, and thawing process seems to be largely attributable to the PUFA contents of sperm plasma membrane (5, 14). In general, the plasma membrane of different mammalian species contains approximately 70% phospholipids, 25% neutral lipids and 5% glycolipids (on molar base) (7).

The role of Ω-3 PUFA on sperm resistance to cooling and storage is controversial (15). In rabbits, the association of dietary Ω-3 fatty acids with vitamins E and C has improved semen quality during semen storage (16). In buffalo, feeding of sunflower oil or sunflower seed has resulted in improvement of the spermatozoa quality (17). In contrast, Castellano et al. (15) reported that adding fish oil to the diet failed to improve the quality of cryopreserved boar sperm. Furthermore, some recent experiments did not find any improvement of stored semen quality when the Ω-3 PUFAs were added to the diet of pigs (18), rabbits (19), and stallions (20). Also, supplementation of boar semen extender with docosahexaenoic acid (DHA)-enriched egg yolk did not increase boar sperm resistance to freezing (21). Taken together, these studies have revealed inconsistent effects of PUFAs on sperm quality when they are used in diet or extender. 

In bull, spermatogenesis lasts for about 2 months (11). Modified ration should therefore be given for 1-2 months to see any effects of diet changes on sperm parameters. Dietary supplementation experiments are time-consuming and costly. Thus, because of incompatible results in previous studies, saving time and reducing cost, the present study was designed to investigate the potential protective effects of Ω-3 PUFAs, added to extender, on bovine semen quality in response to cooling and cryopreservation procedures.

## Materials and Methods

### Reagents and media

This experimental study included ejaculates from five proven fertile bulls, allocated to control and four experimental groups. For group 1, polyethylene glycol (PEG) as a solvent was added alone to the extender, while for groups 2, 3 and 4, different concentration of 1, 2.5 and 5%, respectively, of omega-3 PUFAs (WN pharmaceutical Ltd., BC, Canada) in combination with PEG were added to the semen extender. The basic extender (BX) used in the experiments contained 2.91% sodium citrate, 20% egg yolk and 7% glycerol. In order to make a homogeneous extender and to introduce Ω-3 PUFAs to semen extenders, 5% polyethylene glycol (PEG) had to be added as a solvent. Media were then sonicated for six minutes. 

### Semen collection and processing

The Ethical Committee of Ferdwosi University of Mashhad approved all procedures used in this study. Semen was collected by artificial vagina from five fertile Holstein bulls by conventional method of sampling at the Khorasan Breeding Center, Mashhad, Iran. Immediately after collection, ejaculates were transferred to a water bath at 37˚C and examined for semen volume, color, pH, sperm motility and sperm concentration. Only the ejaculates with more than 70 % motility were selected for further processing. The selected semen samples were divided into five parts and diluted (40×10^6^ sperms/ml) at 37˚C by different media in order to achieve the proper concentrations for control and treatments groups (Table 1). After an equilibration period of three hours at 4˚C, some semen samples were frozen by fast method of freezing and stored at -196˚C in liquid nitrogen for one month, while other semen samples were kept in refrigerator (5˚C) up to 24 or 48 hours.

**Table 1:** Characteristics of diluting media used for control and four treatment groups


Group	Composition of diluting medium

**Control (CTR)**	Basic extender (BX)
**Group 1 (PEG)**	BX + 5% PEG
**Group 2 (LOW)**	BX + 5% PEG + 1% Ω-3 PUFA
**Group 3 (MED)**	BX + 5% PEG + 2.5% Ω-3 PUFA
**Group 4 (HIGH)**	BX + 5% PEG + 5% Ω-3 PUFA


### Experimental design

#### Experiment 1- liquid storage of semen

In experiment 1, the effect of different levels of Ω-3 PUFAs on sperm quality was assessed during storage at 5˚C for 24 and 48 hours, respectively. Sperm aliquots were taken from refrigerator and incubated for five minutes at 37˚C before sperm analyses. 

#### Experiment 2-cryopreservation of semen

In this experiment, the effect of different levels of Ω-3 PUFAs on frozen-thawed sperm quality was assessed. Straws from each sample were thawed at 37˚C for 30 seconds in a water bath and examined.

#### Assessment of sperm motility

Computer aided sperm analysis (HFT-CASA V6.50, Hooshmand Fanavar Tehran Co., Iran) was used for assessment of motility parameters. For evaluation, a 10 μl drop of sample (further diluted to 1×10^7^ spermatozoa/ml with BX) was placed onto a pre-warmed slide, covered with a cover slip of 22×22 mm and studied using a negative contrast-phase optical microscope (×100) (Olympus, Germany) at 37˚C. Five fields of each drop were recorded and processed. This CASA system is based upon the analysis of 25 consecutive digital images obtained from a single field using a camera (Olympus, Germany). Approximately, 200 cells were evaluated per field. Total and progressive motility, different motility classes (A: rapid progressive, B: slow progressive and C: non- progressive), static class (D), curvilinear velocity (VCL), linearity (LIN), average path velocity (VAP), straight-line velocity (VSL) and amplitude of lateral head displacement (ALH) were determined.

#### Assessment of sperm viability

To assess sperm viability, 10 μl of sperm suspension were mixed with 10 μl of eosin solution (0.5 %). Immediately, uncolored sperms were counted under a phase contrast microscope Olympus, Germany to calculate the percentage of sperm viability. Two hundred spermatozoa were evaluated to determine viability.

#### Assessment of sperm morphology

Sperm morphology was examined in smears stained with eosin and nigrosin. The staining solution contained 0.67% eosin Y and 10% nigrosin dissolved in 0.9% sodium chloride in distilled water. Fifty microliter of diluted sperm was mixed with 50 μl of stain and incubated for five minutes. Smears were made on slides and allowed to dry. Slides were mounted and observed under ×400 objective lens of a phase contrast microscope. For each preparation, 200 cells were counted, and the percentages of various defects were enumerated. The morphological defects of acrosome, head, mid-piece and tail were evaluated.

#### Statistical analyses

Each experiment was replicated five times. The statistical analysis was performed using SPSS statistical software version 16 (SPSS Inc., Chicago, IL, USA). Repeated measures ANOVA followed by Bonferroni post-hoc test were conducted to investigate the effects of different levels of Ω-3 PUFAs on sperm quality during the study period. P<0.05 were regarded as statistically significant.

### Results

#### Experiment 1: Liquid storage of semen for 24 and 48 hours

Sperm quality parameters of fresh semen are presented as mean and standard deviation (SD) in table 2. Static class (D) was increased, while all other parameters were decreased over the liquid preservation period in all groups including control. After 24 hours of liquid cold storage, significant decreases (p<0.05) were observed in most quality parameters when compared with fresh semen. Although parameters were decreased during the next 24 hours of preservation, they were not significant.

**Table 2:** Sperm quality parameters of fresh semen collected from five bulls. Data are presented as Mean ± SD


Variable	Mean ± SD

**Total motility%**	81.22 ± 4.87
**Progressive motility%**	67.85 ± 7.26
**Motility classes (A)%**	43.84 ± 9.20
**Motility classes (B)%**	24.01 ± 2.90
**Motility classes (C)%**	13.37 ± 4.14
**Static class (D)%**	24.77 ± 12.13
**Curvilinear velocity (VCL) µm/S**	89.12 ± 27.67
**Linearity (LIN)%**	39.22 ± 4.93
**average path velocity (VAP) µm/S**	54.57 ± 13.35
**Straight-line velocity (VSL) µm/S**	42.16 ± 10.67
**Amplitude of lateral head displacement (ALH) μm**	6.30 ± 1.64
**Viability%**	96.60 ± 3.71
**Normal morphology%**	93.40 ± 1.95


**Fig 1:**
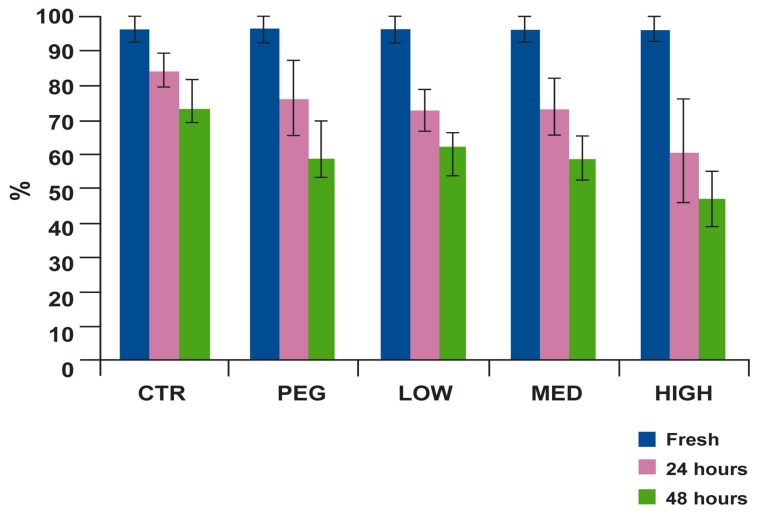
Percentage of viability of bull sperm in fresh, 24 and 48 hours after storage in refrigerator for five treatment groups. During the study period, the percentage of viability in the control was significantly greater than HIGH group (p<0.05).

Different concentrations of omega-3 supplementation did not improve morphology and motility parameters, significantly, during the liquid preservation period. Furthermore, average of viability in group 4 (BX plus PEG and 5% PUFAs) was significantly decreased as compared to control group (p=0.001), which indicates an adverse effect on viability (Fig 1).

#### Experiment 2: cryopreservation of semen

**Fig 2:**
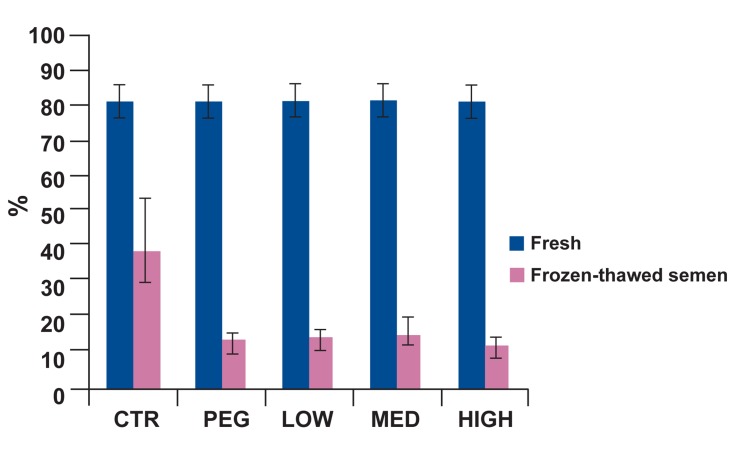
Percentage of total motility in fresh and frozenthawed semen for five treatment groups. At post-thaw, total motility was significantly greater in the control than all other groups (p<0.05).

**Fig 3:**
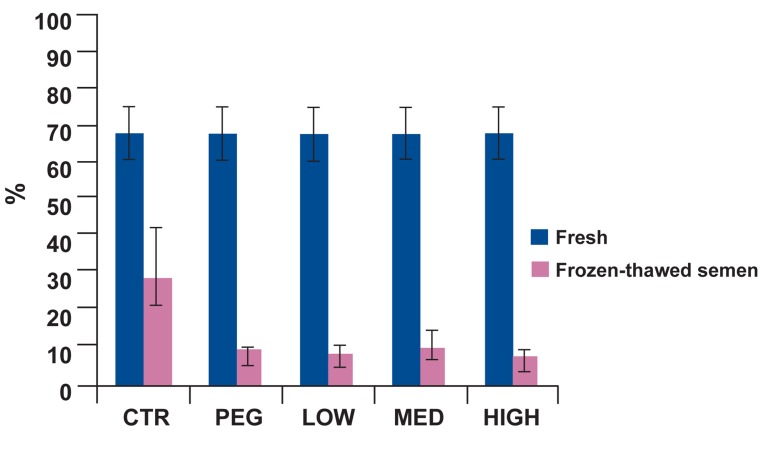
Percentage of progressive motility in fresh and frozenthawed semen for five different treatment groups. In post-thaw samples, percentage of progressive motility was significantly greater in the control than all other groups (p<0.05).

**Fig 4:**
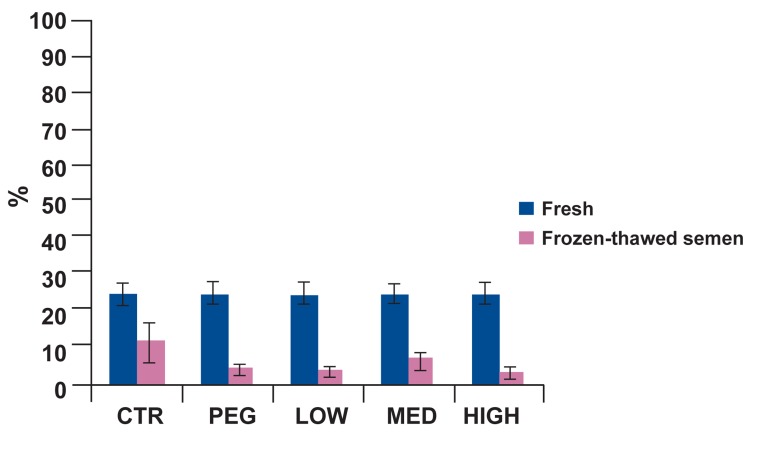
Percentage of motility class (B) in fresh and frozenthawed semen for five treatment groups. At post-thaw, motility class (B) was significantly greater in the control than all other groups (p<0.05).

**Fig 5:**
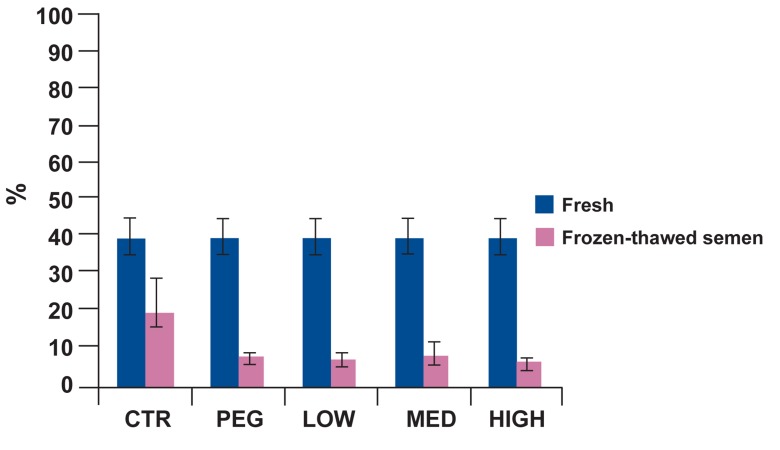
Percentage of linearity in fresh and frozen-thawed semen for five treatment groups. In the post-thaw samples, percentage of linearity in control was significantly greater than all other groups (p<0.05).

**Fig 6:**
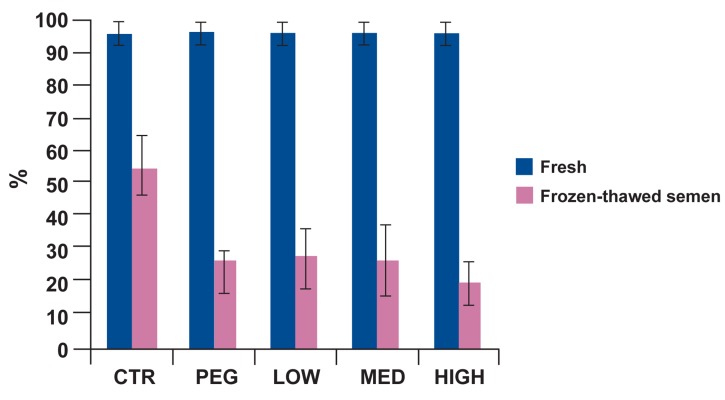
ntage of viability in fresh and frozen-thawed semen for five treatment groups. At post-thaw, Percentage of viability in control was significantly greater than all other groups (p<0.05).

Static class (D) was increased, while other quality parameters were decreased significantly after one month cryopreservation within all studied groups including control as compared with fresh semen (p<0.001).

 motility, progressive motility, motility classes (B), static class (D), linearity and viability in control group were significantly better than treatment groups (Figs 2-6). Post-hoc pairwise comparisons showed that average viability, total motility, progressive motility, motility classes (B) and linearity in control group were significantly greater than all treatment groups (p<0.05). Static class (D) in control group was significantly lower than four treatment groups (p<0.05). 

## Discussion

As expected from previous studies (11), chilling, especially cryopreservation, had a detrimental impact (compared to fresh semen) on sperm characteristics, e.g. motility parameters and viability. Our results showed no remarkable protective effect of PUFAs for any criteria (motility, viability and morphology) over 48 hours of both storage and freeze-thaw procedures.

The role of Ω-3 PUFA, eicosapentaneoic acid (EPA) and docosahexaneoic acid (DHA) on sperm resistance to cooling and freezing procedures is controversial, and may be related to the type of long chain PUFA content (15). Although both positive and negative actions are theoretically possible, the overall effects of PUFAs on fertility are not fully understood. With regard to male fertility, PUFAs are essential substances for male fertility as they give appropriate fluidity to the sperm plasma membrane. (21). To our knowledge, this study is the first attempt to administer Ω-3 PUFAs to bovine spermatozoa *in vitro* in order to evaluate their possible protective effects. Actually, the aim of our study was to determine whether supplementation of semen extender with various concentrations of Ω-3 PUFAs could enhance the thermal resistance of bull semen during cold liquid and frozen storage procedures.

As PUFAs are hydrophobic, a suitable solvent was required. Many industrial products can be categorized as oil-in-water (O/W) emulsions, which consist of small lipid droplets dispersed in an aqueous medium. Polyethylene glycol has the ability to dissolve hydrophobic materials at the lowest concentration. Furthermore, it was the least detrimental effect to sperm motility compared to other solvents such as ethanol and dimethyl sulfoxide (DMSO) (22), and PEG was therefore chosen as the most suitable solvent. Nonetheless, PEG alone had some detrimental effects on sperm quality parameters (group2), and the addition of PUFAs could not attenuate its harmful effects on most of sperm quality parameters.

Results showed that the detrimental effects of cryopreservation were greater than the effects of cold liquid storage on sperm parameters. No improvement of sperm quality was observed after cryopreservation of bull semen following the addition of PUFAs to semen extender.

Addition of DHA enriched egg yolk to the boar semen diluent before freezing did not improve quality of frozen-thawed boar sperm. Various conditions are associated with the low quality of bull sperm after long term cooling storage and cryopreservation (11, 21). After cryopreservation, the level of lipid peroxidation dramatically increases, which indicates cold-shock damage to membranes of sperm, and is consistent with impaired sperm function (4, 15). Long chain PUFAs have been found in the spermatozoa of different species including man, ram and bull. These unsaturated fatty acids result in fluidity of the sperm plasma membrane which is necessary for the membrane fusion events during fertilization. However, long chain PUFAs are attacked by reactive oxygen species (ROS), which initiate a lipid peroxidation cascade that results in deleterious effects on sperm function. Vitamin E, as extracellular antioxidant is able to reverse the negative effect of PUFA supplementation on mammalian spermatozoa (21).

Based on our data, it seems that the addition of Ω-3 PUFAs directly to semen extenders is not effective in protecting the sperm membrane. Instead, they might be supplemented to the diet of bulls in order to modify the fatty acid compositions of sperm and to perform their preventive properties. Several studies have shown that the addition of PUFAs to the diet can influence biosynthetic pathways of both prostaglandin synthesis and steroidogenesis, which are important in reproductive regulation (21). Supplementation of daily boar ration with 3% fish oil increased the DHA content of the spermatozoa, followed by increasing the number of sperm in the ejaculate without any improvement of sperm freezability (18). Feeding sunflower oil improved the post-thawed quality of buffalo bull spermatozoa (17), while dietary DHA supplementation only improved *in vitro* quality of bovine fresh semen without any pronounced effect on frozen-thawed semen (23). Moreover, feeding stallions of marginal fertility with the DHAenriched nutriceutical improved the motion characteristics of their cool-stored semen and the freezability of their sperms (24). Furthermore, the PUFA composition of the cell membranes of the sperm and oocyte is important during fertilization. Signal transduction pathways of fertilization process might be affected by changes in membrane fluidity (21). 

## Conclusion

This study was conducted to investigate possible protective effects of different levels of Ω-3 PUFA in extender on bovine chilled as well as frozen sperm. In contrast to our hypothesis, supplementation of semen extenders with Ω-3 PUFAs did not significantly improve sperm resistance to cooling, especially to cryopreservation. So, it reveals that the addition of Ω-3 PUFAs in bull extender is not a useful method for improvement of bull sperm, and further investigations should be conducted with diet supplementation of different sources of PUFAs in order to convey their protective properties on sperm membranes.
